# A zebrafish model of chordoma initiated by notochord-driven expression of HRASV12

**DOI:** 10.1242/dmm.013128

**Published:** 2013-12-05

**Authors:** Alexa Burger, Aleksandr Vasilyev, Ritu Tomar, Martin K. Selig, G. Petur Nielsen, Randall T. Peterson, Iain A. Drummond, Daniel A. Haber

**Affiliations:** 1 Cancer Center, Massachusetts General Hospital, Charlestown, MA 02129, USA.; 2 Howard Hughes Medical Institute, Chevy Chase, MD 20815, USA.; 3 Division of Nephrology, Massachusetts General Hospital, Charlestown, MA 02129, USA.; 4 Department of Pathology, Massachusetts General Hospital, Boston, MA 02114, USA.; 5 Department of Biomedical Sciences, NYIT COM, Old Westbury, New York, NY 11568, USA.; 6 Cardiovascular Research Center, Massachusetts General Hospital, Charlestown, MA 02129, USA.; 7 Broad Institute, Cambridge, MA 02142, USA.

**Keywords:** HRASV12, Cancer, Chordoma, Drug treatment, Rapamycin, Zebrafish

## Abstract

Chordoma is a malignant tumor thought to arise from remnants of the embryonic notochord, with its origin in the bones of the axial skeleton. Surgical resection is the standard treatment, usually in combination with radiation therapy, but neither chemotherapeutic nor targeted therapeutic approaches have demonstrated success. No animal model and only few chordoma cell lines are available for preclinical drug testing, and, although no druggable genetic drivers have been identified, activation of EGFR and downstream AKT-PI3K pathways have been described. Here, we report a zebrafish model of chordoma, based on stable transgene-driven expression of HRASV12 in notochord cells during development. Extensive intra-notochordal tumor formation is evident within days of transgene expression, ultimately leading to larval death. The zebrafish tumors share characteristics of human chordoma as demonstrated by immunohistochemistry and electron microscopy. The mTORC1 inhibitor rapamycin, which has some demonstrated activity in a chordoma cell line, delays the onset of tumor formation in our zebrafish model, and improves survival of tumor-bearing fish. Consequently, the HRASV12-driven zebrafish model of chordoma could enable high-throughput screening of potential therapeutic agents for the treatment of this refractory cancer.

## INTRODUCTION

Chordoma is a rare, slow-growing tumor that is hypothesized to arise from remnants of embryonic notochord cells. Complete surgical removal of the tumor whenever possible is the standard of care, usually in combination with radiation therapy, because the tumor is known to be highly chemoresistant with few proven systemic therapies available for patients with unresectable disease or distant metastases (Walcott et al., 2012). To date, genetic analyses have shown recurrent allelic losses to be common in this tumor, including the CDKN2A/CDKN2B and PTEN loci in 70–80% of cases (Hallor et al., 2008; Le et al., 2011). Other genes implicated in chordoma include tuberous sclerosis complex (*TSC*) (Lee-Jones et al., 2004), the retinoblastoma (*RB*) tumor suppressor gene (Eisenberg et al., 1997) and *TP53* (Naka et al., 2005; Pallini et al., 2003). In addition, expression of the master transcriptional regulator *T* (Brachyury) is considered pathognomonic of chordoma. *T* gene duplications have been reported in rare cases with genetic predisposition to familial chordoma (Yang et al., 2009), along with amplification of the entire *T* gene locus in some sporadic chordomas (Hallor et al., 2008; Le et al., 2011; Presneau et al., 2011).

However, genetic studies have not revealed mutations that can be targeted therapeutically. Preclinical screening for novel drugs has also been limited by the small number of established chordoma cell lines and by the absence of genetic model systems. Studies of human chordoma samples have suggested activation of the EGFR-PI3K-AKT-mTOR pathway (Han et al., 2009; Presneau et al., 2009; Schwab et al., 2009), and treatment of the chordoma cell line U-CH1 with the mTORC1 and PI3K inhibitor induces apoptosis (Schwab et al., 2009). Clinical trials have been performed using a variety of receptor-tyrosine-kinase inhibitors, including imatinib, sunitinib and EGFR inhibitors, as well as the mTOR inhibitor rapamycin combined with imatinib (Stacchiotti et al., 2009; Walcott et al., 2012). Although partial responses have been observed in some patients, there is a pressing need for high-throughput preclinical models to direct targeted therapies in patients with this relatively rare tumor.

Zebrafish genetic models are well suited to study cancers originating in developmental structures such as the notochord, because stable expression of transgenic constructs is readily accomplished. The transparent fish allow for early detection of abnormally proliferating GFP-tagged cells, even within regions such as the notochord, which is otherwise difficult to visualize. Furthermore, zebrafish models can be easily subjected to high-throughput drug screens (North et al., 2007; White et al., 2011). Here, we define a novel zebrafish model of chordoma, driven by notochord-specific GFP-tagged HRASV12 expression. The fish model is histologically comparable to human chordoma, and has a rapid onset, which is well suited to drug screening. As proof of principle, we demonstrate a partial response to rapamycin, with a delay in the onset of the tumor phenotype and extension of survival.

## RESULTS

### Notochord-specific expression of HRASV12 leads to notochord tumor formation

**Fig. 1.**
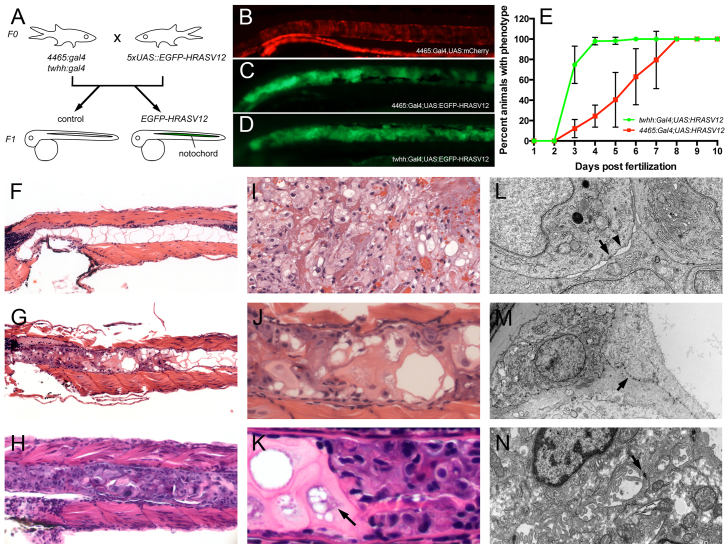
**A novel zebrafish model of chordoma.** (A) Notochord-specific *Gal4* lines (*4465:Gal4* and *twhh:Gal4*) were independently crossed to *UAS:EGFP-HRASV12* heterozygous fish, resulting in the embryos shown in B–D. (B) Control notochord of *4465:Gal4,UAS:mCherry* embryos; (C) notochord of *4465:Gal4;UAS:EGFP-HRASV12*; (D) notochord of *twhh:Gal4;UAS:EGFP-HRASV12. HRASV12* gene transactivation was monitored via GFP in the notochord (C,D). (B-D) Disorganized growth of notochord tissue is evident in C and D compared with a normal ‘stack of coins’ notochord appearance in *4465:Gal4,UAS:mCherry* embryos (B). The abnormal notochord phenotype was evident as early as 3 dpf (data not shown), and progressively increased with age, with 100% of larvae involved by 8 dpf. (B–D) Pictures are representatives from 10 dpf old animals. (E) The phenotype progressed much faster in *twhh:Gal4;UAS:HRASV12* compared with *4465:Gal4;UAS:HRASV12*. (F–N) Histological and ultrastructural examination revealed the presence of a chordoma-like notochord tumor in the transgenic larvae. (F) At 7 dpf, control animals displayed a normal notochord with large vacuolated spaces, thin cytoplasmic septae and bland nuclei. In contrast, *4465:Gal4;UAS:HRASV12* (G,J) and *twhh:Gal4;UAS:HRASV12* (H,K) fish showed a replacement of the notochord by a chordoma-like tumor (compare with an example of human chordoma in I). (L) The tumor cells displayed characteristic desmosomal junctions (arrow) with the formation of ‘windows’ between neighboring cells (arrowhead), which is a common characteristic of human chordomas. In addition, the tumor cells displayed a prominent rough endoplasmic reticulum (ER). (M) The tumor cells often lifted the notochord cells from the basement membrane while still attached to them by numerous desmosomal junctions, which are a part of notochord normal anatomy (arrow). (N) An example of human chordoma showing desmosomal junctions (arrow).

In studying the oncogenic effect of activated HRAS in the zebrafish gut, we expressed N-terminal GFP-tagged HRASV12 using the gut-specific enhancer-trap line *Tg(mü4465_13:Gal4,UAS:mCherry)* (referred to as *4465:Gal4* or 4465; Fig. 1A; supplementary material Fig. S1, left panels). Our expectation was that double-transgenic *4465:Gal4;UAS:HRASV12* zebrafish embryos would display GFP and mCherry co-labeling in early intestinal tissue. Surprisingly, although these fish displayed mild intestinal hyperplasia (supplementary material Fig. S1, right panels), they also rapidly developed prominent notochord malformations. These abnormalities, which were most prominent in the anterior notochord, were characterized by aberrant cellular proliferation, and were present as early as 3 dpf, with 70% penetrance by 7 dpf, 100% penetrance by 8 dpf, and subsequent death of the larvae (Fig. 1 and supplementary material Movies 1–3). This striking, yet unexpected, phenotype presumably results from the fact that the *4465:Gal4* enhancer-trap zebrafish line feature the tiggywinkle hedgehog (*twhh*) minimal promoter to drive *Gal4* transcription. Indeed, *twhh* is prominently expressed in the developing notochord (Du and Dienhart, 2001), and the *4465:Gal4* enhancer-trap lines feature an mCherry-positive notochord during development (control, Fig. 1B). Consequently, double-transgenic *4465:Gal4;UAS:EGFP-HRASV12* embryos potently express the *EGFP-HRASV12* transgene in the notochord, as revealed by GFP fluorescence in the notochord (Fig. 1C).

**RESOURCE IMPACT**

**Background**

Chordoma is a type of bone cancer affecting the spine and base of the skull. These tumors are hypothesized to arise from remnants of embryonic notochord cells. The cancer is known to be highly chemoresistant, with few proven systemic therapies available for cases with unresectable disease or distant metastases. To date, genetic studies have not revealed recurrent mutations that could be targeted therapeutically. Preclinical screening for novel drugs has also been limited by the small number of established chordoma cell lines and the absence of genetic model systems. There is therefore a pressing need for high-throughput preclinical *in vivo* models to direct targeted therapies in individuals with this challenging disease.

**Results**

In this study, the authors describe a novel zebrafish model of chordoma. In this model, tumorigenesis is driven by notochord-specific GFP-tagged HRASV12 expression (HRASV12 is a well-known oncogenic mutation). Using a quantitative GFP-based assay, the authors show that tumors are rapidly induced in live animals in response to HRASV12 expression. Promisingly, the fish model is histologically comparable to human chordoma. As proof-of-principle, the authors demonstrate a partial response to the mTOR inhibitor rapamycin, which has shown some success in clinical trials for the treatment of chordoma. Treatment of the fish model with rapamycin causes a delay in the onset of the tumor phenotype and extension of survival.

**Implications and future directions**

The rapid onset of the tumor phenotype in the zebrafish chordoma model described here would be advantageous for screening for new pharmacological agents that could suppress tumor proliferation or induce tumor cell death. Fish larvae are highly drug-permeable, which, combined with the use of a quantitative GFP assay, lends itself to high-throughput drug-screening protocols. The activity of rapamycin in attenuating the zebrafish phenotype provides further support to its clinical relevance to human chordoma, and sets the stage for a more comprehensive drug-screening strategy. The successful generation of a zebrafish chordoma model using notochord-targeted expression of HRASV12 through the modular UAS/Gal4 system also establishes a general strategy for ectopically expressing other oncogenes implicated in chordoma, including Brachyury.

To confirm that the notochord abnormalities were due to *twhh*-driven *EGFP-HRASV12* expression in notochord cells, we repeated these experiments using *twhh:Gal4* driver transgenics crossed to *UAS:EGFP-HRASV12. twhh:Gal4;UAS:HRASV12* fully and reproducibly recapitulated the notochord phenotype with full penetrance (Fig. 1D). Compared with *4465:Gal4*-driven embryos, the notochord phenotype in *twhh:Gal4*-driven embryos displayed an increased penetrance, with virtually 100% of animals affected as early as 4 dpf (Fig. 1E). Overall, the phenotype progressed much faster in *twhh:Gal4;UAS:HRASV12* animals compared with *4465:Gal4;UAS:HRASV12* (Fig. 1E), although we only observed a significant difference in GFP intensity at 6 dpf (supplementary material Fig. S2). Taken together, these findings establish that notochord-specific HRASV12 expression during zebrafish development causes aberrant proliferation of notochord cells.

### HRASV12-induced notochord tumors are similar to human chordomas

**Fig. 2.**
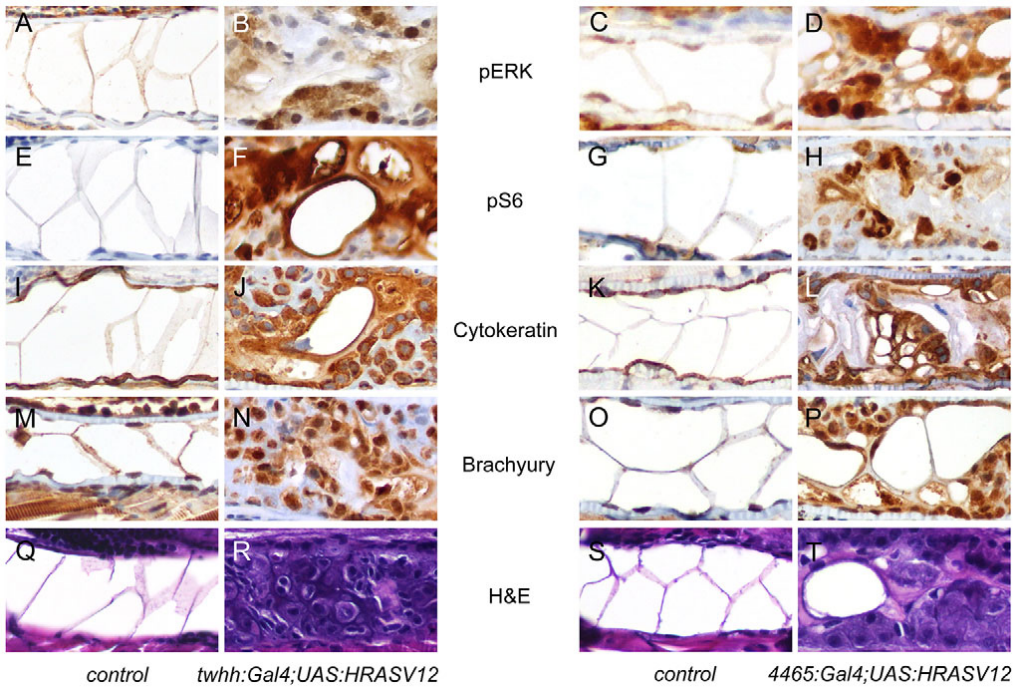
**Immunohistochemical features of the zebrafish notochord tumors.** 7-dpf larvae were examined by immunohistochemical techniques. The left two columns show control and *twhh:Gal4;UAS:HRASV12*, and the right two columns show control and *4465:Gal4;UAS:HRASV12*. (A–D) pERK staining in control embryos (A,C) showed minimal nuclear positivity in the notochord cells. *EGFP-HRASV12* embryos showed focal, strong nuclear and weak cytoplasmic positivity in tumor cells (B,D). (E–H) pS6 staining in control embryos was negative in the notochord (E,G), but the tumor cells showed strong nuclear and weak cytoplasmic positivity. (I–L) Cytokeratin staining showed cytoplasmic positivity in normal notochord cells (I,K) and in the tumor (J,L). (M–P) Brachyury staining demonstrated nuclear and, to a lesser extent, cytoplasmic positivity in normal notochord cells (M,O) and in the tumor (N,P). (Q–T) Corresponding histology of normal notochord (Q,S) and the notochord tumor (R,T).

Because human chordomas are thought to originate from hyperplasia of remnant notochord cells, we investigated the pathology of the observed zebrafish notochord abnormalities that were highly similar to a human chordoma (Fig. 1G,I,J). Compared with control notochords from *4465:Gal4* embryos (Fig. 1F), the malformed notochords from both double-transgenic notochord-*Gal4;UAS:HRASV12* combinations had histological characteristics that were highly similar to those of human chordomas: the developing masses consisted of plump cells with focally prominent nuclear pleomorphism (variation in size and shape) and hyperchromasia (dark staining with hematoxylin). They grew in nests and cords, and focally in a solid pattern (Fig. 1G,H,J,K). The tumor matrix was variable, ranging from dense eosinophilic to myxoid with vacuolated spaces (Fig. 1G,H,J,K), typical of chordoma. We observed large cells with a vacuolated cytoplasm, reminiscent of physaliferous cells of human chordomas (Fig. 1K, arrow). Electron microscopy showed characteristic multifocal desmosomal junctions with formation of ‘windows’ between neighboring cells, similar to those of human chordoma (Fig. 1L–N). In addition, the tumor cells showed positive immunohistochemical staining for the markers used in the diagnosis of chordoma, Brachyury and Cytokeratin, as well as for the signaling molecules pERK and pS6 (Fig. 2A–P). Of note, we could not detect a significant change in the chordoma marker Brachyury prior to the onset of tumor (30 hpf) in the HRASV12-overexpressing embryos compared with controls (supplementary material Fig. S3). The primary distinction between the observed zebrafish notochord tumors and the human chordoma is the rapid phenotype onset in the fish model, compared with the slow-growing human cancer, which typically takes 5 years from onset of symptoms to diagnosis. HRAS mutations have not been reported in human chordomas, and we presume that activation of the proliferative signals might be relatively attenuated in the human disease.

### Rapamycin delays HRASV12-dependent notochord tumor formation and extends survival

A key application of zebrafish tumor models is the ability to screen for chemical compounds that modify the disease phenotype and provide potential therapeutic leads for treating the human disease. Although only limited studies have been performed in cultured chordoma cell lines, these have been shown to respond to the mTORC1/PI3K inhibition (Schwab et al., 2009). To test the feasibility of *in vivo* drug application and screening using our chordoma zebrafish model, we treated HRASV12-expressing embryos as well as their control siblings with rapamycin, LY294002 and bpV(HOpic), a pTEN inhibitor. Larvae were incubated at the previously established concentrations of 10 μm rapamycin, 15 μm LY294002 and 10 μm bpV(HOpic), which were first added at 24 hpf to avoid potential developmental effects of the chemicals (Makky et al., 2007), with additional drug replaced at 3 and 5 dpf.

**Fig. 3.**
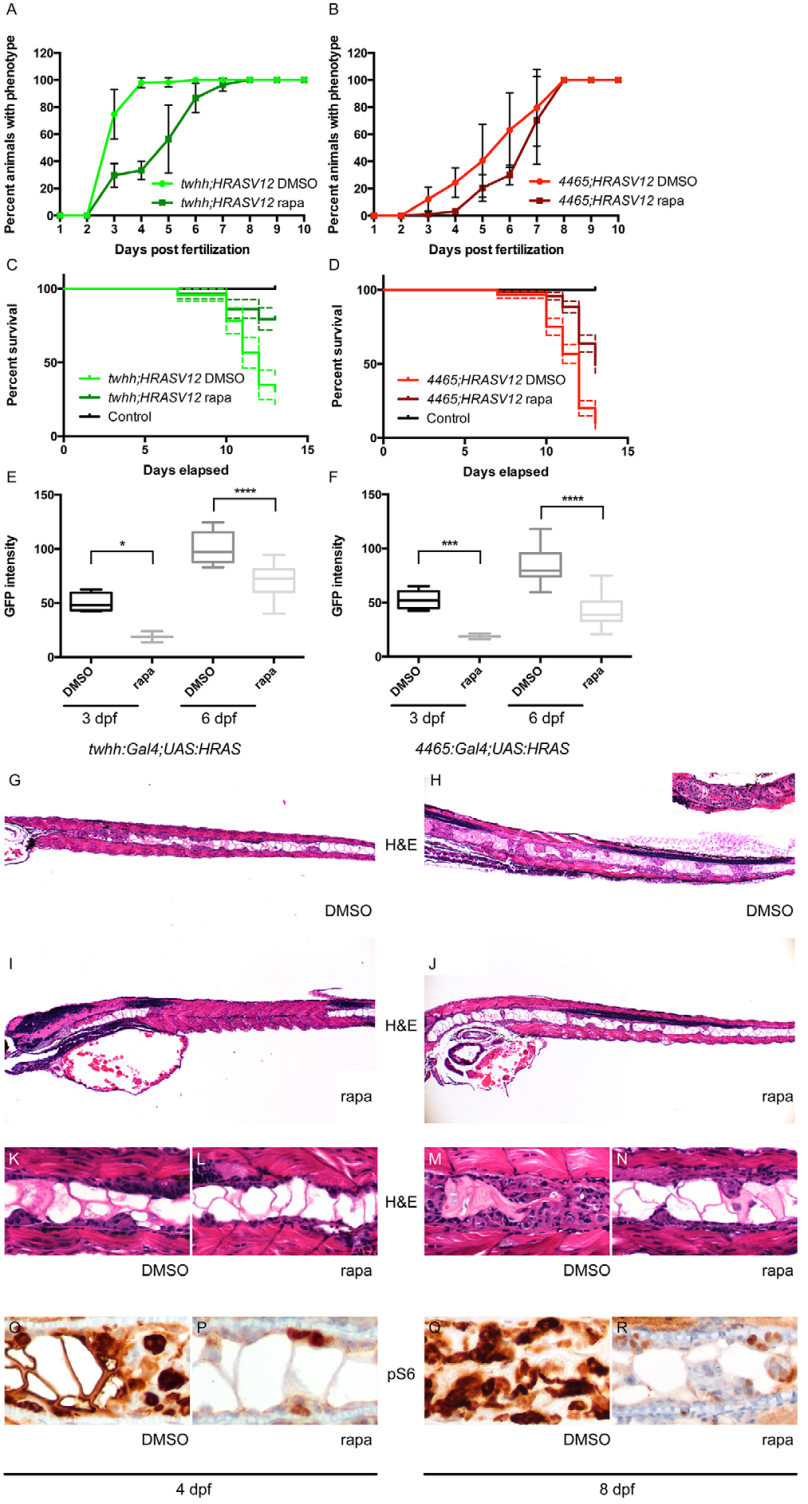
**Rapamycin inhibits notochord tumor progression.** (A,B) Rapamycin treatment delayed the onset of the zebrafish notochord tumor in both *twhh:Gal4;UAS:HRASV12* (A) and *4465:Gal4;UAS:HRASV12* (B) fish. (C,D) The delayed onset of the phenotype correlated with a better survival of rapamycin-treated animals compared with DMSO-treated controls [*twhh:Gal4;UAS:HRASV12* (C), *P*<0.0001 and *4465:Gal4;UAS:HRASV12* (D), *P*<0.0001]. The dashed lines represent 95% confidence intervals. (E,F) Simple measurement of average GFP intensity in the proximal notochord showed a significant difference between DMSO- and rapamycin-treated *twhh:Gal4;UAS:HRASV12* (E) and *4465:Gal4;UAS:HRASV12* (F) zebrafish at 3 and 6 dpf. *P=0.0281; ***P=0.006; ****P≤0.0001. (G–N) The decrease in average GFP intensity was presumably due to reduced tumor growth as shown by H&E staining. (G,K) At 4 dpf, *twhh:Gal4;UAS:HRASV12* transgenic embryos already showed a significant tumor formation presenting as solid growth anteriorly and as a more nested growth posteriorly. (I,L) Rapamycin-treated embryos showed only minimal tumor formation at 4 dpf, even anteriorly. (H,M) At 8 dpf, the tumor growth progressed compared with at 4 dpf, and variably involved the entire length of the notochord with severe involvement of the anterior notochord (inset in H), whereas rapamycin-treated embryos showed a much milder, predominantly nested pattern of growth (J,N). Immunohistochemistry showed a focal, but less intense, staining for pS6 in rapamycin-treated animals (P,R) compared with DMSO-treated controls (O,Q).

In both *twhh:Gal4;UAS:HRASV12* and *4465:Gal4;UAS:HRASV12* embryos, rapamycin treatment caused a significant delay in tumor initiation and progression (Fig. 3A,B) compared with LY294002 and bpV(HOpic), which did not show any consistent phenotype changes and were therefore excluded from further studies (supplementary material Fig. S4). In *twhh:Gal4;UAS:HRASV12* embryos, rapamycin treatment decreased the percentage of animals displaying the notochord phenotype from 100% to 30% at 4 dpf. In *4465:Gal4;UAS:HRASV12*, it almost completely inhibited phenotype onset up to 4 dpf. Ultimately, rapamycin did not prevent tumor formation, because tumors developed in 100% of fish by 7 dpf in *twhh:Gal4;UAS:HRASV12* and by 8 dpf in *4465:Gal4;UAS:HRASV12*. Rapamycin treatment did not alter the expression of the *Gal4* transgene, as assessed by the amount of the mCherry marker (supplementary material Fig. S5). We also quantified tumor growth by measuring average GFP intensity in the anterior notochord in live *twhh:Gal4;UAS:HRASV12* and *4465:Gal4;UAS:HRASV12* transgenics. A steady increase in GFP intensity was evident as tumors progressed from 3 to 6 dpf (Fig. 3I,J), which was suppressed in rapamycin-treated embryos (Fig. 3I,J). Light microscopic analysis of *twhh:Gal4;UAS:HRASV12* larvae supported this conclusion, revealing a reduced tumor mass with more nested discontinuous tumor growth, as opposed to the solid proliferation seen in DMSO-treated fish (Fig. 3K–N). In *4465:Gal4;UAS:HRASV12* transgenics, which display a milder phenotype compared with direct *twhh:Gal4-driven* transgenics, we found animals with near-complete inhibition of tumor growth (data not shown). Moreover, rapamycin-treated larvae survived significantly longer than DMSO-treated controls (Fig. 3E,F; *P*<0.0001 for both *twhh:Gal4;UAS:HRASV12* and *4465:Gal4;UAS:HRASV12*); this effect correlated with reduced pS6 staining in rapamycin-treated embryos at early (4 dpf) and later (8 dpf) stages in development compared with DMSO-treated controls, indicating effective mTOR inhibition (Fig. 3O–R).

## DISCUSSION

We report the first *in vivo* genetic model for chordoma, a rare cancer of notochordal remnant cells with limited therapeutic options. Notochord-specific expression of HRASV12 in zebrafish, using available stable transgenic lines, generates a reproducible tumor phenotype that should greatly expand the tools available to study this malignancy. Although mutations of RAS family members are not common in chordoma, activation of HRAS can mimic activation of downstream signaling pathways driven by cell surface receptors, such as EGFR, which has been dominantly implicated in chordoma (Dewaele et al., 2011; Launay et al., 2011; Ptaszyński et al., 2009; Weinberger et al., 2005). However, limitations of this model include the fact that inhibitors of targets acting upstream of HRAS (such as EGFR) will not be readily testable in this model. Indeed, the proliferative rate of the tumor, one of the major differences between the zebrafish model and human chordoma, might be a reflection of direct versus indirect activation of the RAS pathway. Nonetheless, RAS-driven tumor models have proven useful in defining the cell of origin of a rare tumor type, defining biological properties of the malignant cells, and potentially screening for lineage-specific pathways that could be targeted therapeutically. The *UAS:EGFP-HRASV12* transgene that we employed here has been successfully used to transform melanocytes, resulting in tumors with immunological, histological and molecular features of human melanoma (Santoriello et al., 2010), and is comparable to previously reported RAS-driven zebrafish tumor models affecting liver (Nguyen et al., 2011) and pancreas (Davison et al., 2008).

The HRASV12-expressing zebrafish tumors are highly similar to the human chordoma, based on histological, ultrastructural and immunohistochemical features. The zebrafish died around 14 dpf, presumably from reduced motility and altered feeding behavior, before we could observe tumor invasion of the basement membrane or distant metastases. The development of conditional transgenic lines, or tumor transplantation studies, could help refine the biological properties that can be tested in this model. Of note, the phenotypes that we observed with two different HRASV12 constructs were virtually identical, but did show some differences in the timing of tumor onset, aggressiveness and response to rapamycin. These differences are likely to result from increased transgene expression in the *twhh:Gal4* transgenic line, consistent with the measured GFP signal intensity.

The rapid onset of the tumor phenotype in our zebrafish chordoma model is well suited for screening for new pharmacological agents that can suppress tumor proliferation or induce tumor cell death. The affected larval stage is highly drug permeable and the application of a quantitative GFP assay lends itself to high-throughput screening protocols. Such screens have been successfully accomplished using zebrafish, leading to clinical trials (North et al., 2007; Goessling et al., 2011). As proof of principle, we show that the mTOR inhibitor rapamycin results in significant changes in tumor phenotype. The activity of rapamycin in attenuating the zebrafish phenotype lends further support to its candidacy based on human chordoma cell lines (Schwab et al., 2009), and sets the stage for a more comprehensive drug screening strategy. It still needs to be determined whether this rapamycin effect has therapeutic value. We observed the greatest drug effect following application at early time points; however, more effective drugs or drug combinations might lead to pronounced effects even at more advanced stages of chordoma tumorigenesis in this model. In fact, the robustness of the HRAS-driven phenotype could allow high-throughput screens, and be subject to validation in more physiologically driven models.

We also tested the potential role of PI3K signaling in our zebrafish chordoma model using the PI3K inhibitor LY294002 and the PTEN inhibitor bpV(HOpic), but did not observe any inhibitory or activating effects on tumor growth, possibly due to PI3K-independent activation of mTOR by the *RAS* oncogene (Shaw and Cantley, 2006). However, it is still possible that a combined inhibition of PI3K and mTOR will produce a much stronger inhibitory effect on tumor growth even in the absence of a specific PI3K inhibitor response (Schwab et al., 2009). It remains to be tested whether a similar relationship can be demonstrated in our zebrafish chordoma model. A more likely possibility is that a constitutively active RAS is sufficient to drive mTOR activation independent of PI3K through redundant mechanisms, such as the RAF-MEK-ERK pathway.

The successful generation of a zebrafish chordoma model using notochord-targeted expression of HRASV12 through the modular *UAS/Gal4* system establishes a screening tool for testing additional potential molecular targets, and also establishes a general strategy for ectopically expressing other oncogenes implicated in chordoma, including Brachyury, in this system (Hallor et al., 2008; Le et al., 2011).

## MATERIALS AND METHODS

### Zebrafish maintenance and transgenic strains

Adult zebrafish were maintained and embryos were obtained according to standard fish husbandry protocols (Westerfield, 2000) in accordance with Massachusetts General Hospital animal protocols. Strains included: *Tg(mü4465_13:Gal4,UAS:mCherry)* (Distel et al., 2009); *Tg(5XUAS:eGFP-HRASV12)* (Santoriello et al., 2010); *Tg(twhh:Gal4)* (described elsewhere). The *Tg(mü4465_13:Gal4,UAS:mCherry)* line was generated by an enhancer-trap screen (http://www.helmholtz-muenchen.de/en/idg/groups/neuroimaging/lines_distel/main.html).

### Drug treatment

Embryos were either drug- or vehicle-treated (1% DMSO). In total, three doses of rapamycin (final concentration 10 μM; R0395, Sigma-Aldrich, St Louis, MO), LY294002 (final concentration 15 μM; 440202, Calbiochem, Millipore Corporation, Billerica, MA) or bpV(HOpic) (final concentration 10 μM; 203701, Calbiochem, Millipore Corporation, Billerica, MA) were given at 24 hpf, 3 dpf and 5 dpf.

### Gross morphology and histological analysis

Transgenic zebrafish were observed under the Olympus MVX10 stereomicroscope for gross notochord morphology and pictures taken with the Olympus DP72 camera. mCherry and GFP intensities were measured in the anterior notochord using ImageJ software (NIH). Embryos were fixed at different developmental stages in 4% paraformaldehyde at 4^°^C overnight. Fixed embryos were washed in PBS with 0.1% Tween 20 and afterwards dehydrated in alcohol, cleared in xylene and infiltrated with paraffin. Tissue sections (4 μm thick) from paraffin-embedded tissue blocks were placed on charged slides, deparaffinized in xylene, rehydrated through graded alcohol solutions and stained with hematoxylin and eosin (H&E). Immunohistochemical studies for cytokeratin, brachyury, pERK and pS6 were performed according to the manufacturers’ protocol using anti-cytokeratin (961, Abcam, Cambridge, UK), anti-brachyury (sc-20109, Santa Cruz, Dallas, TX), anti-pERK (4376, Cell Signaling, Danvers, MA) and anti-pS6 (2211, Cell Signaling, Danvers, MA). Images were taken with the Canon EOS Rebel T2i digital camera, mounted with a custom optical adapter onto the Olympus BX40 microscope.

### Electron microscopy

8-dpf zebrafish larvae were placed into electron microscopy fixative (2.5% glutaraldehyde, 2.0% paraformaldehyde, 0.025% calcium chloride in a 0.1 M sodium cacodylate buffer, pH 7.4) and allowed to fix overnight at 4^°^C. The fixative was replaced with cacodylate buffer and the zebrafish were stored at 4^°^C until further processing in a Leica Lynx™ automatic tissue processor. The larvae were post-fixed with osmium tetroxide, en bloc stained with 2.0% uranyl acetate dehydrated in a graded ethanol series, embedded in pure epoxy resin and polymerized overnight at 60^°^C. 1 μm thick sections were cut using glass knives and a Sorvall MT-1 (Dupont) ultramicrotome, and floated on water droplets on glass slides. The slides were dried in a humidity chamber on a warm hot plate. Toluidine blue stain (0.5% toluidine blue in aqueous 0.5% sodium borate) was pipetted over the sections and placed onto the hot plate until a slight gold rim could be seen around the stain droplet. The sections were rinsed in a stream of distilled water, dried, cover slipped and examined by light microscopy. Tissues representing the notochord lesions were chosen, and the blocks trimmed accordingly. Thin sections were cut using a diamond knife and an LKB 2088 ultramicrotome and placed on copper grids. Sections were stained with lead citrate and examined in a FEI Morgagni transmission electron microscope. Images were captured with an Advanced Microscopy Techniques 2K digital CCD camera. Global contrast was corrected in Photoshop (Adobe Systems Inc.).

### Statistical analysis

Kaplan-Meier curves were computed using the survival distribution of each group. The log-rank test was used to compare significant differences in death rates between drug- or vehicle-treated fish with a 95% confidence interval. Raw survival data are shown in supplementary material Table S1. For comparison of mCherry and GFP intensity in drug- or vehicle-treated fish, the unpaired *t*-test was used. All data are representatives of at least two different biological replicates.

### Live imaging

Transgenic zebrafish were immobilized in low-melting-point agarose and imaged in time-lapse confocal stacks using a Zeiss LSM5 or Nikon C2 confocal microscope as described (Vasilyev and Drummond, 2012). Time-lapse movies were assembled using ImageJ software (NIH).

## Supplementary Material

Supplementary Material
