# Supplementary material for: A zebrafish model of chordoma initiated by notochord-driven expression of HRASV12
Source: Dis Model Mech. 2013 Dec 5;7(7):907–13. doi: 10.1242/dmm.013128 (PMC4073279; doi:10.1242/dmm.013128)
Supplement: Supplementary Material [file supp_7_7_907__index.html]

A zebrafish model of chordoma initiated by notochord-driven expression of HRASV12 — Supplementary Material 

# A zebrafish model of chordoma initiated by notochord-driven expression of HRASV12

## DMM013128 Supplementary Material

**Files in this Data Supplement:**

- **Supplementary Material**
