# Supplementary material for: A zebrafish model of chordoma initiated by notochord-driven expression of HRASV12
Source: Dis Model Mech. 2013 Dec 5;7(7):907–13. doi: 10.1242/dmm.013128 (PMC4073279; doi:10.1242/dmm.013128)
Supplement: Supplementary Material [file supp_7.7.907_DMM013128.pdf]

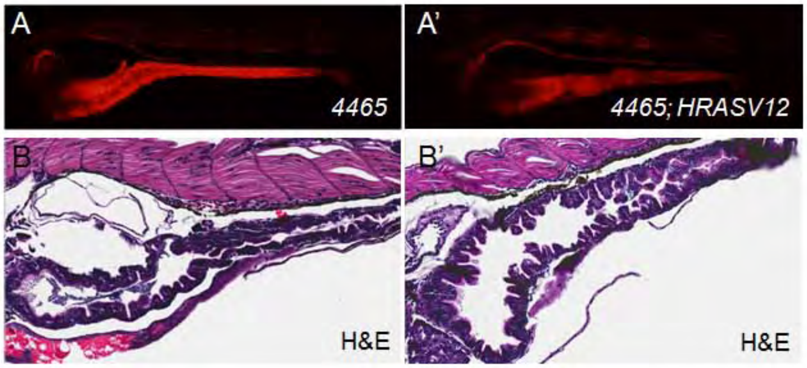

Supplemental Figure S1.
(A) Gross morphology of Tg(mü4465_13:Gal4,UAS:mCherry) using fluorescent microscopy. mCherry is expressed in the gut, kidney, and notochord. (B) H&E staining. (A’, B’) Same as in (A-B), but double transgenic 4465:Gal4;UAS:HRASV12.

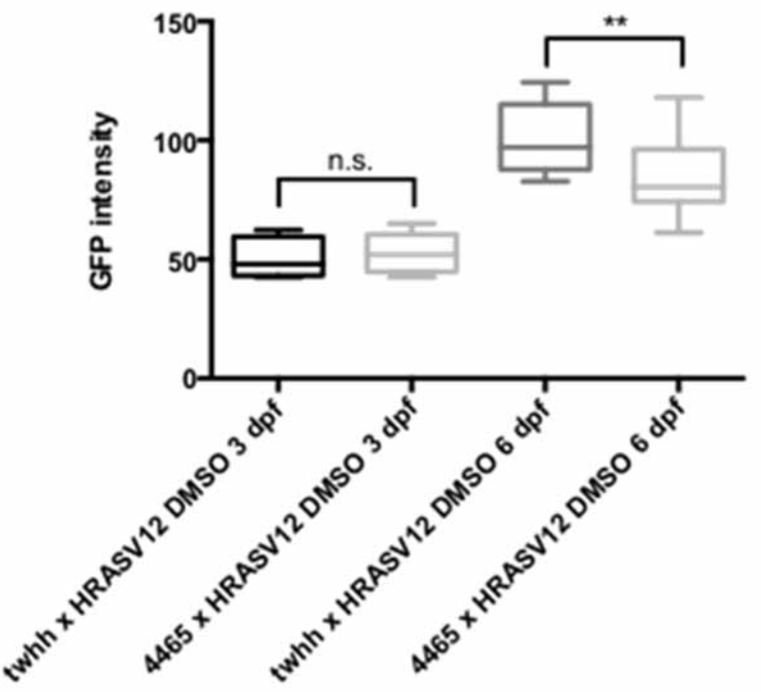

Supplemental Figure S2.
GFP intensity is similar at 3 dpf in 4465;HRASV12 and twhh;HRASV12 embryos, but increases significantly at 6 dpf in twhh;HRASV12 embryos compared to 4465;HRASV12 embryos (unpaired t-test; $p=0.0025$). Each time point and condition includes $n>4$ animals.

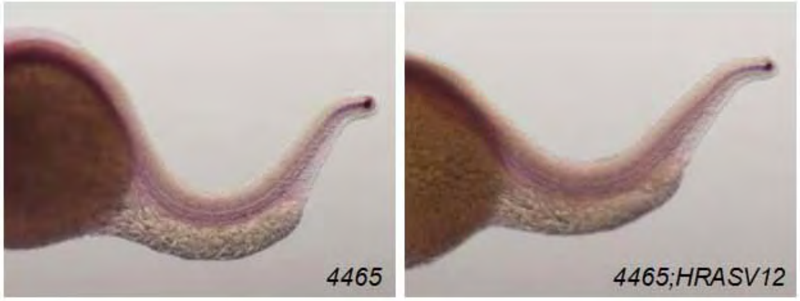

Supplemental Figure S3.
No tail (ntl; Brachyury) in situ hybridization of 30 hpf 4465;Gal4 and 4465;HRASV12 embryos. No significant change in ntl-expression upon HRASV12-overexpression, suggesting that Brachyury is independent of HRASV12. In situ hybridization was performed according to standard protocols for zebrafish embryos.

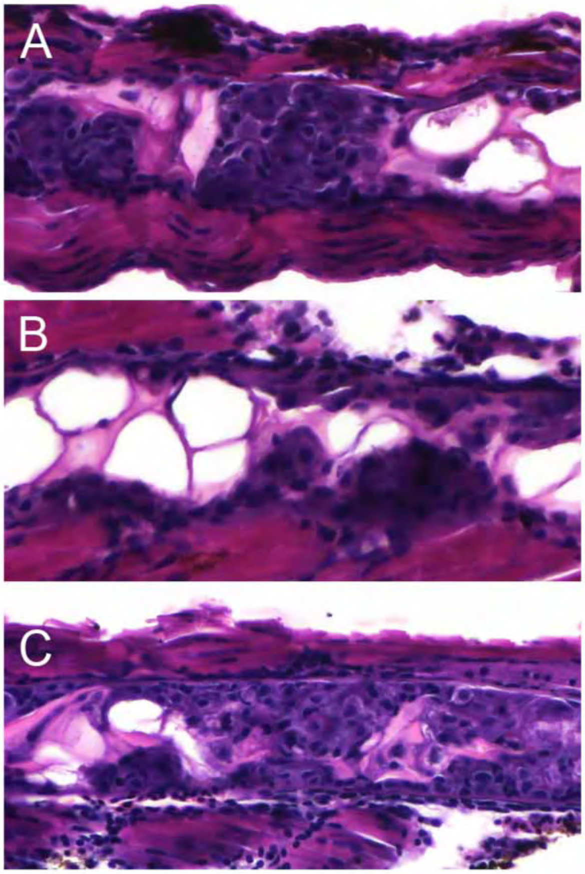

Supplemental Figure S4.
(A) 8 dpf old twwh:HRAS embryos treated with 1% DMSO (control) show the typical tumor phenotype. (B) 8 dpf old twwh:HRAS embryos treated with 15 µM LY294002 with slightly reduced tumor load; however, this effect was inconsistent and LY294002 treatment did not result in improved survival (data not shown). (C) 8 dpf old twwh:HRAS embryos treated with 10 µM bpV(HOpic). The observed tumor load was similar to that in control fish.

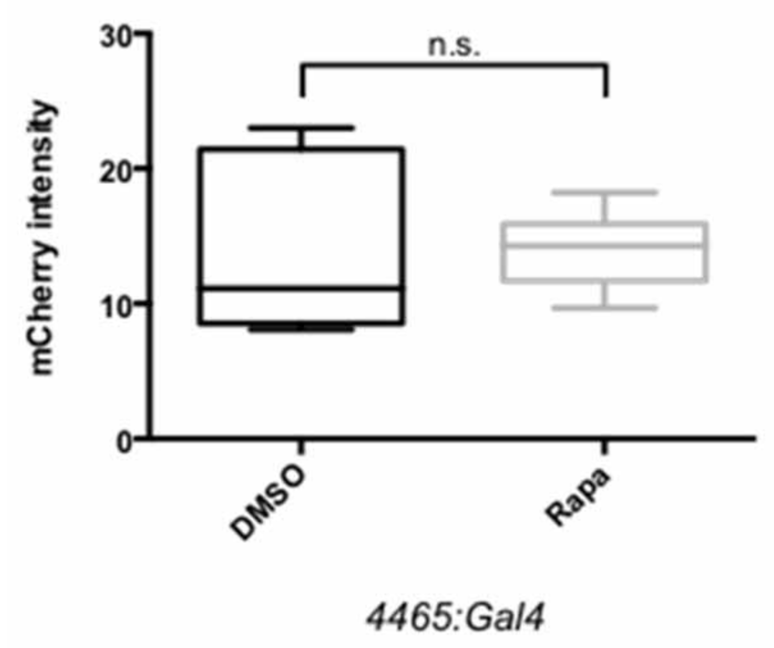

Supplemental Figure S5.
mCherry intensity in the anterior notochord of rapamycin- vs. DMSO-treated 4465:Gal4 larvae is not significantly different (unpaired t-test; p=0.9239). mCherry intensity was measured at 7 dpf in n=9 animals in rapamycin- and n=5 in DMSO-treated fish.

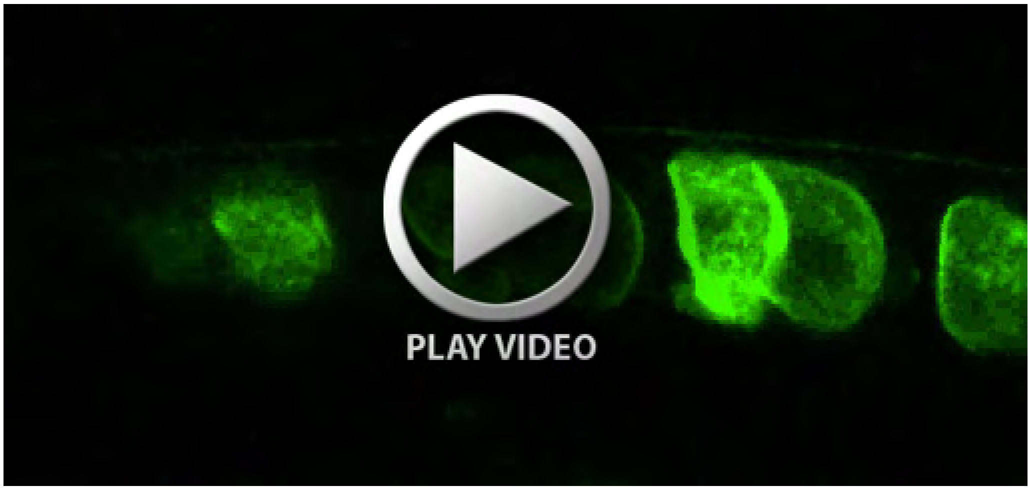

Supplemental Movie S1. Notochord cell proliferation in a twhh:Gal4;HRASV12 transgenic embryo.
Notochord cells were imaged by time lapse confocal microscopy at 36 hpf in twhh:Gal4;UAS:HRASV12 transgenic fish. Arrowheads mark cell division events. Images represent z-projection of 5 confocal slices (10um total thickness). Frame interval = 20min, number of frames = 35.

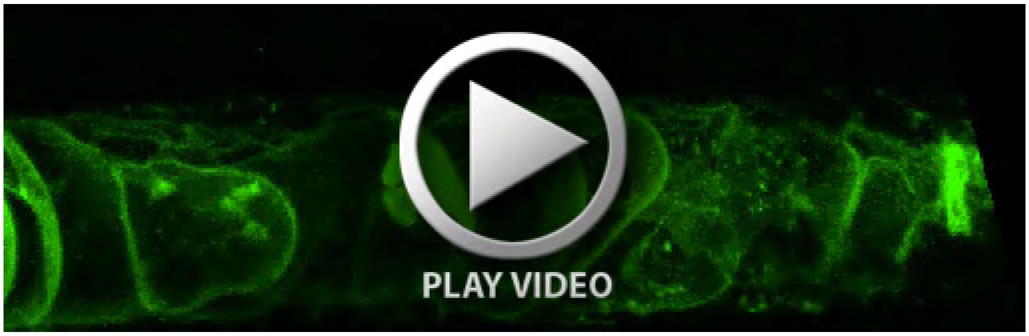

Supplemental Movie S2. Notochord cell proliferation in a 4465:Gal4;HRASV12 transgenic embryo.
Notochord cells were imaged by time lapse confocal microscopy at 3 dpf in 4465:Gal4;UAS:HRASV12 transgenic fish. Numerous cell division events are evident. Images represent z-projection of the entire thickness of the notochord. Frame interval = 20min, number of frames = 43.

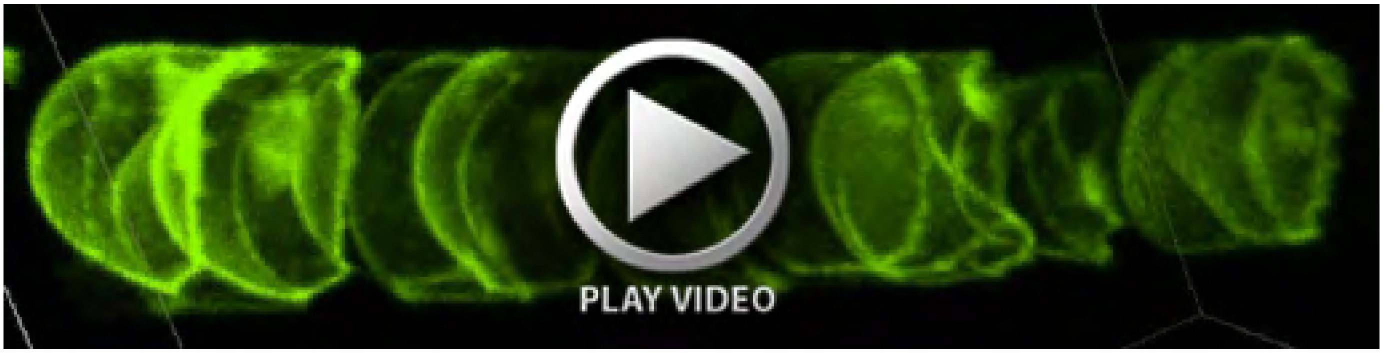

Supplemental Movie S3. Lack of cell proliferation in a 4465:Gal4;UAS:YFP transgenic embryo.
Notochord cells were imaged by time lapse confocal microscopy at 3 dpf in 4465:Gal4;UAS:YFP transgenic fish. No cell division events are seen. Images represent z-projection of the entire thickness of the notochord. Frame interval = 15 min, number of frames = 49. The Tg(UAS:YFP) line will be described elsewhere.

Supplemental Table 1. Raw survival data of the Kaplan-Meier analysis.
We used Prism software to determine survival rates in rapamycin-treated vs. control embryos, in which each row represents one subject at a given time point (days elapsed = x value). The Y value stands for one individual embryo which is "1" when the embryo died at the specified time, and "0" when the embryo's data was censored at that time.

Download Table S1
